# The characteristics and experiences of parents accessing prescribed safer supply in BC, 2020—2021

**DOI:** 10.1186/s12954-026-01468-0

**Published:** 2026-05-10

**Authors:** Katherine Hogan, Celeste Macevicius, Taija McLuckie, Jenny McDougall, Bernie Pauly, Karen Urbanoski, Brittany Barker

**Affiliations:** 1 Canadian Institute of Substance Use Research, University of Victoria, 2300 McKenzie Ave, Victoria, BC V8N 5M8 Canada; 2 School of Public Health and Social Policy, University of Victoria, PO Box 1700 STN CSC, Victoria, BC V8W 2Y2 Canada; 3 School of Nursing, University of Victoria, PO Box 1700 STN CSC, Victoria, BC V8W 2Y2 Canada; 4 Faculty of Health Sciences, Simon Fraser University, Blusson Hall, 8888 University Drive, Burnaby, BC V5A 1S6 Canada

**Keywords:** Prescribed safer supply, Parenting, Harm reduction, Substance use, Child welfare system, British Columbia, Canada

## Abstract

**Objectives:**

In 2020, British Columbia implemented prescribed safer supply (PSS) to reduce harms associated with the toxic drug supply. Evidence is emerging on these programs; however, no studies to date have focused on exploring the experiences of parents accessing or attempting to access PSS, including differences between parents and non-parents in reported barriers.

**Methods:**

This mixed-methods study involved a secondary analysis of data collected for an evaluation of PSS, informed by an ecological model. We conducted descriptive analysis of barriers to care, using data from a cross-sectional survey with people who had received or were seeking PSS (n = 353), and a thematic analysis of data from interviews with a subset of participants who were parenting children under age 19 (n = 16), focused on exploring parents’ experiences of PSS.

**Results:**

Among 353 recruited, 31.4% of participants reported having a child under age 19. All participants reported barriers to PSS; however, parents were more likely to report being too busy compared to non-parents (41.4% vs. 25.4%, *p* = 0.003). Ecological themes describing parents’ experiences of PSS reflected: (1) unique interpersonal motivations for accessing PSS, such as to participate in children’s lives and protect them from potential harms and (2) parenting-associated policy barriers, including fear of child welfare reports and competing time demands.

**Discussion:**

Parents reported unique interpersonal motivations and policy challenges when accessing PSS. These findings highlight the need to address stigmatizing, punitive responses to families experiencing substance use and support approaches that are parent-centered and inclusive of the family across a continuum of care.

## Introduction

In 2016, the Public Health Officer of British Columbia (BC) declared a public health emergency due to the exponential rise in toxic drug fatalities, driven by the proliferation of illicitly-manufactured fentanyl in the street supply [1]. Since the declaration the crisis has continued, claiming over 13,000 lives between 2016 and 2023 [1]. In 2020, the BC government approved the Risk Mitigation Guidance (RMG) as a response to the dual public health emergencies of COVID-19 and an increasingly toxic drug supply. The RMG supported clinicians to prescribe alternatives including pharmaceutical-grade opioids (e.g., hydromorphone), stimulants (e.g., dextroamphetamine), and benzodiazepines (e.g., alprazolam) to replace the toxic unregulated supply, known as a form of prescribed safer supply (PSS) [2].

The toxic drug supply crisis has had devastating and intergenerational impacts on families and kinship networks. Previous research has identified unique barriers and facilitators for mothers trying to access substance use services and treatment, with little research focusing on fathers and gender diverse parents [3, 4]. Barriers parents experience include internalized stigma, substantiated concerns about (re)involvement of child welfare services or other punitive responses (e.g., forced treatment), judgement from service providers and family. Additional barriers include fear of “failing” treatment, unwillingness to be separate from children and family during intensive treatment, transportation challenges, financial demands, time constraints, and limited availability of relevant services [5–7]. Facilitators included motivations to be a good mother, having trusting relationships with service providers and family, assistance with transportation and childcare, collaborative care models, family-oriented programming, trauma-informed care, and parenting-specific supports [4, 5, 7]. In Canada, while parental drug use itself is not considered a cause for child apprehension, this rationale is used frequently [6, 8]. Parental barriers to care are influenced by social and structural determinants of health and compounded for those experiencing intersecting marginalized identities (e.g., Indigenous people, ethnic and sexual minorities) [6, 7]. Parents who use drugs and have had their child apprehended experience a range of negative impacts, including a heightened risk of overdose, increased drug use, poorer mental health, and decreased motivation in treatment [4].

There are significant gaps in research about the experiences of parents trying to access support for unregulated substance use, particularly for medication-based interventions (e.g., opioid agonist treatment) and populations beyond women in the perinatal period [3]. Indeed, given the extreme stigma associated with parenting and substance use and the surveillance of parents who use drugs by child welfare and healthcare services, parents remain a hidden population in substance use research. To address this gap, we undertook a secondary analysis of survey and interview data to explore differences in the experiences of parents and non-parents who accessed or tried to access a PSS under RMG. In this study, we refer to all people who have children as parents but recognize that not all individuals identify as parents or are actively parenting.

## Methods

This secondary analysis is part of a larger mixed methods evaluation of the implementation and impacts of the RMG in BC [9]. Details of our study design and methodology have been previously described in detail [10]. In brief, the present study uses a mixed-methods design involving survey and interview data conducted by trained research associates, including people who use(d) drugs. We undertook a participatory approach to all study activities with peers (i.e., people who use(d) drugs) participating in study design, instrument development and piloting survey/interview questions, recruitment, data collection, analysis, interpretation, and reporting, which we have discussed elsewhere in detail [11]. Recruitment posters and flyers were distributed by peers and through drug user organizations and networks, as well as via social media, harm reduction services, OAT and primary care clinics, and non-profit organizations. Surveys were administered between October 2020-October 2021 and interviews from November 2020-December 2021. Data were collected primarily by phone or in-person in accordance with COVID-19 guidelines. Eligible participants were 19 or older, had used unregulated drugs in the past 6 months, and had received a safer supply prescription or were actively seeking one (i.e., were trying to get a prescription or were planning to do so in the next 2 weeks). We used SPSS version 30.0.0.0 (172) to run bivariate chi-square tests comparing experiences of parents and non-parents with PSS on prescription receipt, medication experiences, non-fatal overdoses, and barriers to PSS. Participants were asked to identify barriers to PSS from a list of 23 that was co-created with people who use(d) drugs. Barriers were collapsed to 12 dichotomous variables (yes vs. no; not mutually exclusive).

We conducted semi-structured interviews with a subset of survey participants (n = 54). Participants were purposively sampled to ensure representation by gender, region, and prescription type. Parenting status was not specifically targeted. For the present study, we included all interview participants who reported being a parent in the survey as the inclusion criteria for this secondary parenting analysis (n = 16). Interviews lasted 1 hour, were recorded, and transcribed verbatim. Although our interview guide did not ask specific questions about parenting, we posed overarching questions about motivations, facilitators, barriers, and impacts related to PSS. Parenting-specific responses emerged to these questions in multiple interviews. This secondary analysis relied on the coding framework developed for the larger evaluation and analysis previously reported [10]. We therefore conducted a thematic analysis of content related to parenting and having a child, informed by the ecological model [12, 13]. KH and CM read all transcripts from parenting participants (n = 16), CM thematically analyzed parenting content, KH organized across the ecological model levels and shared early analysis with BB, BP, TM, and JM who refined themes together. The ecological model sensitized us to consider factors at the intrapersonal, interpersonal, organizational, community, and policy levels [13]. This approach aligns with previous research identifying multi-level influences on parents’ experiences of substance use services [5, 7, 14].

Study activities were approved by the University of Victoria Research Ethics Board (#20—0293).

## Results

### Quantitative Findings

Of the 353 survey participants, 31.4% reported children under age 19 (see [10] for a comprehensive description of this survey sample). Half (50.5%) of parents and 62.2% of non-parents were men, 38.3% and 67.3% were white, 61.7% and 32.7% were BIPOC, 57.5% and 52.7% were stably housed, 69.5% and 81.6% resided in larger urban centers, 45.9% and 40.9% did not complete high school, and 63.1% and 59.9% reported being single. We have chosen not to further disaggregate ethnicity beyond BIPOC (Black, Indigenous, and other People of Colour). This was an intentional decision grounded in a harm reduction approach to research and reporting. Specifically, further racial disaggregation, where not directly tied to the research question, can contribute to stigmatizing or deficit-based interpretations and reinforce surveillance or inequitable policy responses toward racialized communities. This approach aligns with Indigenous, Black, and critical scholarship on race, substance use, and structural vulnerability, which cautions against the routine foregrounding of race in ways that risk harm without clear analytic or community benefit [15–19].

Among parents, 9.0% reported having full-time care of their children and the majority (65.8%) reported children were living with family members. Most (68.5% of parents and 74.2% of non-parents) had accessed PSS. Compared to non-parents, parents were significantly more likely to report being too busy as a barrier to PSS (41.4% vs. 25.4%, p = 0.003). No other significant differences were found between parents and non-parents, including ability to access desired medication, having a sufficient dosage to prevent withdrawal, or experiencing an overdose (Table 1). Many barriers were commonly reported by both parents and non-parents, including mistrust of healthcare providers, past negative interactions and feeling unsafe in clinics, beliefs that PSS will not be able to meet needs, lack of knowledge of where to access PSS, perceived costs, and concerns surrounding COVID-19.

**Table 1:** Experiences of parents and non-parents accessing or trying to access PSS

	Non-Parents	Parents	χ^2^
	(n = 209)	(n = 111)	
Received any prescription (n = 320)			χ^2^(1) = 1.171, *p* = .279
No	54 (25.8)	35 (31.5)	
Yes	155 (74.2)	76 (68.5)	
Type of prescription, n (%)^1`^			
Opioids	134 (64.1)	65 (58.6)	
Stimulants	42 (20.1)	19 (17.1)	
Benzodiazepines	6 (2.9)	2 (1.8)	
No prescription	54 (25.8)	35 (31.5)	
Able to get desired medication (n = 227)^2^			χ^2^(1) = 1.368, *p* = .242
No	42 (27.5)	15 (20.3)	
Yes	111 (72.5)	59 (79.7)	
Dosage high enough for withdrawal (n = 225)^2^			χ^2^(1) = .549, *p* = .459
No	92 (60.1)	47 (65.3)	
Yes	61 (39.9)	25 (34.7)	
Overdose past two weeks (n = 320)			χ^2^(1) = .745, *p* = .388
No	187 (91.2)	97 (88.2)	
Yes	18 (8.8)	13 (11.8)	
Reported barriers			
Covid-19 measures n = 320			χ^2^(1) = 2.154, *p* = .142
No	127 (60.8)	58 (52.3)	
Yes	82 (39.2)	53 (47.7)	
Fear of, or past experiences of, stigma in health care (n = 320)			χ^2^(1) = .013, *p* = .911
No	74 (35.4)	40 (36.0)	
Yes	135 (64.6)	71 (64.0)	
Safety concerns when accessing health care (n = 320)			χ^2^(1) = 1.259, *p* = .262
No	126 (60.3)	74 (66.7)	
Yes	83 (39.7)	37 (33.3)	
Desired PSS medication not available (n = 320)			χ^2^(1) = . 204, *p* = .651
No	94 (45.0)	47 (42.3)	
Yes	115 (55.0)	64 (57.7)	
Previous negative reaction from a health care provider (n = 320)			χ^2^(1) = .015, *p* = .902
No	137 (65.6)	72 (64.9)	
Yes	72 (34.4)	39 (35.1)	
Lack of information about PSS (n = 320)			χ^2^(1) = 1.504, *p* = .220
No	153 (73.2)	74 (66.7)	
Yes	56 (26.8)	37 (33.3)	
PSS prescription cost (n = 320)			χ^2^(1) = 1.316, *p* = .251
No	166 (79.4)	94 (84.7)	
Yes	43 (20.6)	17 (15.3)	
Being too busy for PSS (n = 320)			χ^2^(1) = 8.776, *p* = .003
No	156 (74.6)	65 (58.6)	
Yes	53 (25.4)	46 (41.4)	
Health concerns (n = 320)			χ^2^(1) = .084, *p* = .772
No	109 (52.2)	56 (50.5)	
Yes	100 (47.8)	55 (49.5)	
Don’t think PSS will work (n = 320)			χ^2^(1) = 1.004 *p* = .307
No	157 (75.1)	89 (80.2)	
Yes	52 (24.9)	22 (19.8)	
Mistrust of health care services (n = 320)			χ^2^(1) = .102, *p* = .749
No	143 (68.4)	74 (66.7)	
Yes	66 (31.6)	37 (33.3)	
Lack of privacy in health care services (n = 320)			χ^2^(1) = 1.502, *p* = .220
No	160 (76.6)	78 (70.3)	
Yes	49 (23.4)	33 (29.7)	

^1^Not mutually exclusive (proportions do not sum to 100%)

^2^Only respondents who reported having accessed PSS received this item

### Qualitative Findings

Interviews were diverse with respect to gender, ethnicity, and housing stability. Two main ecological themes were identified: (1) Parents have unique interpersonal motivations for accessing prescribed safer supply (2) Parents experience specific policy and regulatory barriers to accessing prescribed safer supply.

#### Parenting-specific interpersonal motivations in seeking PSS

Some participants identified parenting-related motivations as reasons to pursue or stay on PSS. One motivation identified was improving the quality of the role parents had in their children’s lives: *“I’d like to get back to a point in my life where I can be a functioning part of my daughter’s life”* (participant 3827). PSS was seen as a pathway to improve stability and therefore support more active engagement with their children.

Another motivation was that PSS was viewed as providing separation and therefore protection from the toxicity of the street supply. Parents acknowledged that accessing the unregulated drug market could negatively impact their children: *“I overdosed twice. My son saved me once […] But again, like it takes a whole family. Like I mean, kids are already in it”* (participant 2333). Further participants expressed a need to have ways to avoid these harms: *“I don’t want to die over this. […] I don’t want to leave that legacy for my kids”* (participant 2524). PSS was seen as one way to prevent harm, including children being exposed to a parent in withdrawal, *“I don’t ever want… my son to see me sick. Ever”* (participant 2565). Together, these quotes underline the intergenerational impacts of the toxic drug crisis and parents’ motivation to protect their children from these harms.

#### Parenting-specific policy and regulatory barriers to PSS

Some participants attributed barriers to parenting when trying to access or stay on PSS. One parent described fear that disclosing their substance use and access to PSS would lead their healthcare provider to report them to child welfare services as unfit to parent. They linked fears to past negative experiences of being engaged in substance use treatment: “*I slipped and said I was on methadone to a social worker […] She kind of went from there and decided that she’d better open up a file […] it was just like ‘Really? Because I’m being honest? So what you want me to do is lie to you?’ So that’s why I don’t trust anybody. I just have a hard time trusting anybody*” (participant 2890). Another emphasized how a lack of consideration for children and system involvement can create barriers for parents *“Ah, I think we need to make room for children, um, without the fear of social services coming in”* (participant 2333).

Parents also described the challenge of balancing work and family responsibilities with having their PSS medications dispensed daily at the pharmacy. This participant had their prescription cut off illustrating how a lack of attention and support to parents’ competing commitments and dispensation regulations can act as a barrier to staying on PSS medications: *“I was so busy working and family and stuff that I had missed two days [of dispensations]”* (participant 2565).

## Discussion

Families across BC continue to be impacted by the toxic drug crisis. We found that parents experienced many policy barriers when trying to access PSS. In the survey, parents were more likely to report being too busy to access a prescription than non-parents. This was corroborated by interview findings that parenting responsibilities impeded participants’ ability to access PSS and could result in discontinuation of prescriptions. Parents highlighted that many barriers are the result of regulatory policies and procedures that exist within the current systems of care. Parenting-related time demands have likewise been identified as a barrier and potential factor for disengagement from care in existing research [5, 7]. Other barriers parents reported included fears of drug use stigma from providers, the threat of child apprehension, and a lack of child-inclusive substance use services, highlighting the significant impact of current health and legal policies on parents who use drugs. These barriers have also been reported by parents trying to access other substance use services such as treatment, underlining the need for systems-wide change [5, 7, 20].

Notably, parents also had unique interpersonal motivations for pursuing PSS; namely, to take a more active role in their children’s lives and protect children from harm. This desire to better children’s lives and keep children safer has been noted as a facilitator for engaging in substance use treatment [5]. Further, previous research among mothers who use drugs has reported engaging harm reduction strategies including utilizing family supports for childcare and using substances outside the home and not alone to reduce potential harms to children [21]. This suggests addressing interpersonal factors, including parent–child relationships, may be an important consideration in PSS interventions [5]. Emerging research on PSS programs found that personal connection among providers and participants were a facilitator to access and care [22]. Non-stigmatizing care, respectful treatment, and the availability of wraparound services at PSS programs such as support in obtaining, social assistance and child tax benefits, childcare, housing, and food should be extended to family networks to reduce parenting-related barriers and support families [22].

Finally, we observed that half of parents in the survey identified as men, yet the vast majority of research focuses on mothers who use drugs [3, 21]. Paternal needs in substance use and child welfare services are often overlooked, and this invisibility within services can further isolate fathers from children, fracture existing familial networks, and prevent fathers from achieving their substance use and parenting goals [20].

Our preliminary findings highlight a gap in the literature, and more data are needed to explore the unique factors impacting parents’ access to safer supply and treatment. Future research is needed to explore how parents’ current custody status impacts potential barriers and facilitators to substance use services and safer supply.

## Limitations

The survey sample is limited by the non-random sampling approach that primarily relied on connecting with participants by phone, which may have resulted in an under sampling of those who were the most marginalized. All data were self-reported and prone to potential biases in recall and social desirability. We attempted to minimize these potential biases by engaging peers across the province in helping develop, pilot, and administer the survey. Due to small sample size, we did not stratify parents based on custody status which may influence reported barriers. We were unable to report on the interview sample characteristics because of small sample sizes. Barriers reported by participants may also be impacted by ethnicity differences that are not explored within these analyses. The secondary analysis of qualitative data was valuable in highlighting that parenting has multiple intersections with PSS. However, these findings should be interpreted with caution as we did not include parenting-specific questions in our study instrument and thus we were limited to analyzing parenting-related experiences that were independently raised.

## Conclusion

The intergenerational impact on families ten years into the toxic drug crisis continues. These findings highlight some of the challenges as well as motivations that parents who use drugs, including both fathers and mothers, experience when trying to access PSS and other substance use services. We recommend prioritizing parent-centered goals and echo calls for research, policy and programming that takes the entire family network into account [7].

## Data Availability

The datasets generated and analyzed during the current study are not publicly available to protect individual privacy but are available from the corresponding author on reasonable request.
